# Structural Characterisation of TetR/AcrR Regulators in *Streptomyces fildesensis* So13.3: An In Silico CRISPR-Based Strategy to Influence the Suppression of Actinomycin D Production

**DOI:** 10.3390/ijms26104839

**Published:** 2025-05-19

**Authors:** Karla Leal, Juan Machuca, Humberto Gajardo, Matías Palma, María José Contreras, Kattia Nuñez-Montero, Álvaro Gutiérrez, Leticia Barrientos

**Affiliations:** 1 Facultad de Ingeniería, Instituto de Ciencias Aplicadas, Universidad Autónoma de Chile, Temuco 4780000, Chile; karla.leal@uautonoma.cl (K.L.); m.palma16@ufromail.cl (M.P.); maria.contreras@uautonoma.cl (M.J.C.); 2 Facultad de Ciencias de la Salud, Instituto de Ciencias Biomédicas, Universidad Autónoma de Chile, Temuco 4780000, Chile; juan.machuca@cloud.uautonoma.cl; 3 Facultad de Ciencias de la Salud, Instituto de Ciencias Aplicadas, Universidad Autónoma de Chile, Temuco 4780000, Chile; 4 Laboratory of Integrative Biology (LIBi), Millennium Institute on Immunology and Immunotherapy, Centro de Excelencia en Medicina Traslacional (CEMT), Scientific and Technological Bioresource Nucleus (BI-REN), Universidad de La Frontera, Temuco 4810296, Chile; a.gutierrez05@ufromail.cl

**Keywords:** Antarctic *Streptomyces*, biosynthetic gene clusters, CRISPR-based activation, molecular dynamics simulation

## Abstract

The growing threat of antimicrobial resistance has intensified the search for new bioactive compounds, particularly in extreme environments such as Antarctica. *Streptomyces fildesensis* So13.3, isolated from Antarctic soil, harbours a biosynthetic gene cluster (BGC) associated with actinomycin D production, an antibiotic with biomedical relevance. This study investigates the regulatory role of TetR/AcrR transcription factors encoded within this biosynthetic gene cluster (BGC), focusing on their structural features and expression under different nutritional conditions. Additionally, we propose that repressing an active pathway could lead to the activation of silent biosynthetic routes, and our in-silico analysis provides a foundation for selecting key mutations and experimentally validating this strategy. Expression analysis revealed that TetR-279, in particular, was upregulated in ISP4 and IMA media, suggesting its participation in nutrient-dependent BGC regulation. Structural modelling identified key differences between TetR-206 and TetR-279, with the latter containing a tetracycline-repressor-like domain. Molecular dynamics simulations confirmed TetR-279’s structural stability but showed that the S166P CRISPy-web-guided mutation considerably affected its flexibility, while V167A and V167I had modest effects. These results underscore the importance of integrating omics, structural prediction, and gene editing to evaluate and manipulate transcriptional regulation in non-model bacteria. Targeted disruption of TetR-279 may derepress actinomycin biosynthesis, enabling access to silent or cryptic secondary metabolites with potential pharmaceutical applications.

## 1. Introduction

Antimicrobial resistance is an imminent crisis, with several bacterial pathogens considered “critical priorities” by WHO [1,2]. WHO included mitigating antimicrobial resistance as a major focus in its 2019–2023 work programme [3]. This emergency requires new sources and molecules with antimicrobial properties, as traditional methods often rediscover known compounds. Research in extreme environments offers opportunities for discovering novel antimicrobial compounds [4,5]. The Antarctic Territory, with its cold, dry climate, high UV radiation, and high soil salt concentration, represents such an environment [6,7]. Here, actinobacteria have been studied for novel bioactive molecules due to their adaptation to extreme conditions, involving unique metabolic pathways that can be analysed through metabolomics or genomics [8,9]. Advanced sequencing technologies have revealed new bacterial genome precursors for novel compound synthesis [10,11]. However, most biosynthetic gene clusters (BGCs) remain silenced under laboratory conditions, prompting exploration of activation strategies [10,11].

Núñez-Montero et al. [12] conducted a study with a *Streptomyces fildesensis* So13.3 strain isolated in Antarctica. Sequencing and analysis of the complete 9.47 Mb genome revealed 42 predicted BGCs and 56 putative clusters, constituting approximately 22% of the total genome content. Notably, many of these clusters (11 out of 42 BGCs and 40 out of 56 putative BGCs) showed no similarity to other known BGCs. In a subsequent study conducted by Núñez-Montero et al. [13], our group grew strain So13.3 under various nutrient and elicitation conditions, such as the addition of lipopolysaccharide (LPS), sodium nitroprusside (SNP), and co-culture. Metabolomes obtained by HPLC-QTOF-MS/MS were analysed using molecular networking, linking BGCs identified in the genome with metabolites detected by MS/MS. The results revealed the presence of an actinomycin BGC in strain So13.3, which was corroborated by structural matches with actinomycin D according to genomic analysis. This finding was supported by HPLC-MS/MS spectral analyses of crude organic extracts from the Antarctic strain *Streptomyces fildesensis* So13.3 grown under different culture conditions. A peak corresponding to a potential actinomycin D analogue was observed both under basal conditions and in elicitation treatments. Besides this cluster, the strain has shown the capability to generate a broad spectrum of bioactive metabolites—1171 in total—including several analogues with minor yet functionally significant alterations in their core structures. Nevertheless, these secondary metabolites are not directly linked to growth or reproduction, so their biosynthetic pathways usually remain dormant and are only activated in response to specific environmental signals [14]. In this regard, CRISPR technology has been suggested as a groundbreaking tool in bacteria, as it allows for the precise activation of silent biosynthetic gene clusters, thus enabling the production of secondary metabolites that are typically not expressed [15]. As part of our strategy, we propose to inhibit metabolic pathways related to basal production, with the goal of inducing a metabolic reorganisation that promotes the activation of silent pathways. In this context, the actinomycin D cluster stands out as a promising candidate for targeted activation [16].

Genetic analysis of the antinomycin D cluster reveals different regulatory genes [17], including the TetR/AcrR family transcriptional regulator. TetR/AcrR family members contain an N-terminal DNA-binding motif (HTH) and a C-terminal ligand-recognition domain [18]. The C-terminal domain can identify compounds similar to those transported by target transporters [19]. TetR proteins monitor the cellular environment and regulate genes associated with antibiotic production, osmotic stress, efflux pumps, multidrug resistance, metabolic modulation, and pathogenesis [20,21]. Inhibition of TetR/AcrR family factors within the actinomycin D cluster could contribute to metabolic modification of So13.3 to increase production of new antibiotic metabolites [22]. The CRISPR/Cas9 gene editing method, specifically the pCRISPR-Cas9-scalingD system described by Tong [23], could be used for this inhibition. Given the limited knowledge of TetR/AcrR family regulators within the actinomycin D biosynthetic gene cluster, this study focuses on their structural and dynamic characterisation to support targeted CRISPR-based inhibition strategies. Building on previous evidence confirming the presence of an actinomycin D BGC in *Streptomyces fildesensis* So13.3 and the detection of related metabolites under specific culture conditions, we performed RT-PCR to evaluate the expression of the TetR/AcrR transcription factor under the same nutritional conditions used in the prior metabolomic study. This allowed us to explore the regulatory behaviour of the cluster in response to environmental cues. We hypothesise that repression of this constitutively active pathway could promote the activation of silent biosynthetic routes. Our in-silico analysis provides a rational framework for selecting functional mutations and paves the way for future experimental validation.

## 2. Results

### 2.1. Structural Characterisation and Refinement of TetR Transcription Factors in the Actinomycin D Biosynthetic Cluster of Streptomyces fildesensis So13.3

In the genome of *Streptomyces fildesensis* So13.3 (GenBank accession: CP048835.1), a BGC potentially linked to actinomycin D production was discovered using antiSMASH. This cluster contains two genes that encode putative transcription factors (TFs) of the TetR family, specifically the AccR subtype: TetR-206 (Protein ID: QNA70898.1), with 206 amino acids, and TetR-279 (Protein ID: QNA70921.1), with 279 amino acids. To investigate the structural features of these TFs, BLAST 2.16.0 searches were conducted against the PDB, but no crystallised structures were identified. As a result, homology modelling was performed using AlphaFold, which retrieved UniProt entries A0A5R9MB50 for TetR-206 and A0A5R9M760 for TetR-279. Structural models were created, and their confidence scores were evaluated to pinpoint areas of low structural quality requiring refinement. Further inspection of protein features shows that although both TFs are annotated as TetR/AcrR, TetR-206 presents only an HTH-DNA-binding motif, which is common in TetR transcription factors and other TF families. In contrast, TetR-279 has a specific tetraciclyn repressor-like C terminal domain, as identified by InterProScan in their respective UniProt entries. Previous findings highlight the potential of TetR-279 as the primary regulator of actinomycin D BGC expression, as its C-terminal domain could bind and respond to metabolic stimuli as required.

**Figure 1:**
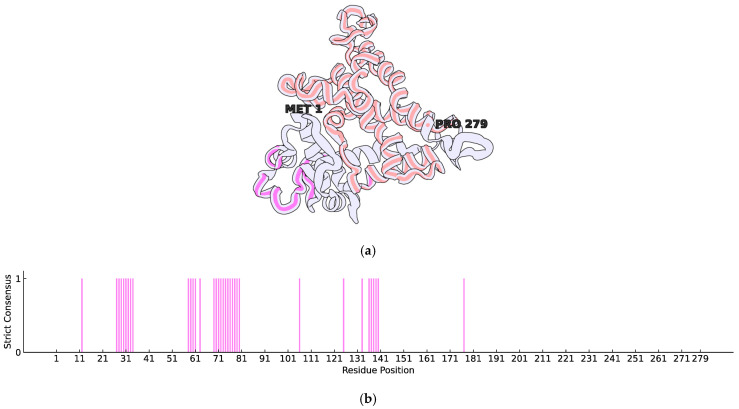
Three-dimensional representative structure of the putative TetR/AcrR protein after 200 ns of molecular dynamics. (**a**) Representative three-dimensional structure of the predicted domains within TetR-279. Regions highlighted correspond to predicted functional structures: residues in orange indicate the tetracycline repressor TetR C-terminal domain, as identified by InterProScan via UniProt; residues in pink represent DNA-binding residues predicted by DP-Bind using the strict consensus criterion. The overall structure is rendered in cartoon style to depict secondary structure elements, while the highlighted regions are shown as backbone traces. The first (N-terminal) and last (C-terminal) residues are labelled for spatial reference. The image was generated using Protein Imager (https://3dproteinimaging.com). (**b**) Strict consensus prediction of DNA-binding residues obtained using the DP-Bind server. The binary values represent residues where all three classifiers (SVM, KNN, and PNN) concurred in a positive prediction. Pink bars indicate positions of agreement among the classifiers.

To improve the structural reliability of these regions, loop refinement was performed using the GalaxyWeb Server, resulting in optimised models. In both cases, the low-confidence regions—according to AlphaFold’s scoring system—were primarily loops near each protein’s N-terminal. The refined structures were used as initial conformations for all-atom MD simulations with GROMACS. Furthermore, to assess whether these transcription factors have membrane-associated functions, SignalP 5.0 was employed to predict the presence of signal peptides. The analysis confirmed the absence of signal sequences, indicating that both proteins are likely cytoplasmic and not directly involved in membrane interactions. Considering that TetR-279 was functionally annotated as having only a C-terminal domain related to ligand binding, and no HTH-DNA-binding motif was reported, we assessed its potential to bind DNA based on sequence-derived features using the DP-Bind webserver (https://lcg.rit.albany.edu/dp-bind, accessed on 30 June 2024). The results indicate that this transcription factor possesses several residues predicted to participate in DNA binding, despite lacking a canonical N-terminal HTH motif (Figure 1b). Most of these predicted residues are located near the N-terminal region, forming clusters around positions 27–34, 58–63, and 69–80. In contrast, the tetracycline repressor TetR C-terminal domain, as defined by InterProScan, spans residues 103–262. Although one DNA-binding residue cluster (positions 136–140) overlaps with this domain, the structural context suggests that these regions occupy distinct spatial locations, likely corresponding to different functional roles—namely, DNA interaction versus ligand binding (Figure 1a).

### 2.2. Nutritional Regulation of TetR-279 Suggests Its Role as a Key Activator of the Actinomycin D Biosynthetic Cluster

**Figure 2:**
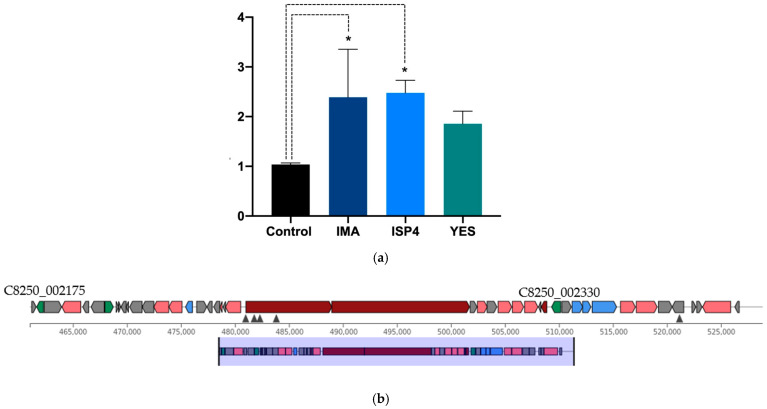
Gene expression analysis revealed a significant upregulation of TetR-279 in IMA and ISP4 media, indicating nutrient-dependent activation of the actinomycin D cluster. (**a**) Relative expression levels of the TetR/AcrR transcription factor within the actinomycin D cluster across three different nutritional environments: IMA (blue bar), ISP4 (light blue bar), and YES (green bar). A significant increase in gene expression was observed in IMA and ISP4 media compared to the M2 (dark bar) control (*, *p* < 0.05). The APRT gene served as an internal reference for normalisation. The analysis was conducted using three biological replicates and two analytical methods per condition. (**b**) Summary of transcription factors identified within the actinomycin D biosynthetic gene cluster. Both proteins belong to the TetR/AcrR family, which is commonly associated with transcriptional repression in response to environmental or metabolic signals.

In order to assess the potential involvement of the TetR-279 transcription factor in the regulation of the actinomycin D biosynthetic gene cluster under specific nutritional conditions, gene expression analysis was conducted using different culture media. The results showed a significant increase in TetR-279 expression in IMA and ISP4 media compared to the control condition M2 (*p* < 0.05), suggesting an association between the activation of this transcription factor and the induction of the BGC in these environments (Figure 2a). In contrast, no significant differences in TetR-279 expression were observed in YES medium, indicating that this condition does not promote BGC activation through this regulator (Figure 2b and Table 1). Relative expression levels were normalised using the adenine phosphoribosyltransferase (APRT) gene as an internal control. Data represent three biological replicates and two technical replicates per experimental condition. Altogether, these findings support the potential role of TetR-279 as a key regulator of actinomycin D biosynthesis in response to specific nutritional cues and suggest that it could serve as a promising candidate for targeted activation of this BGC.

### 2.3. Structural Effects of Point Mutations in TetR-279 via Molecular Dynamics Simulation

**Figure 3:**
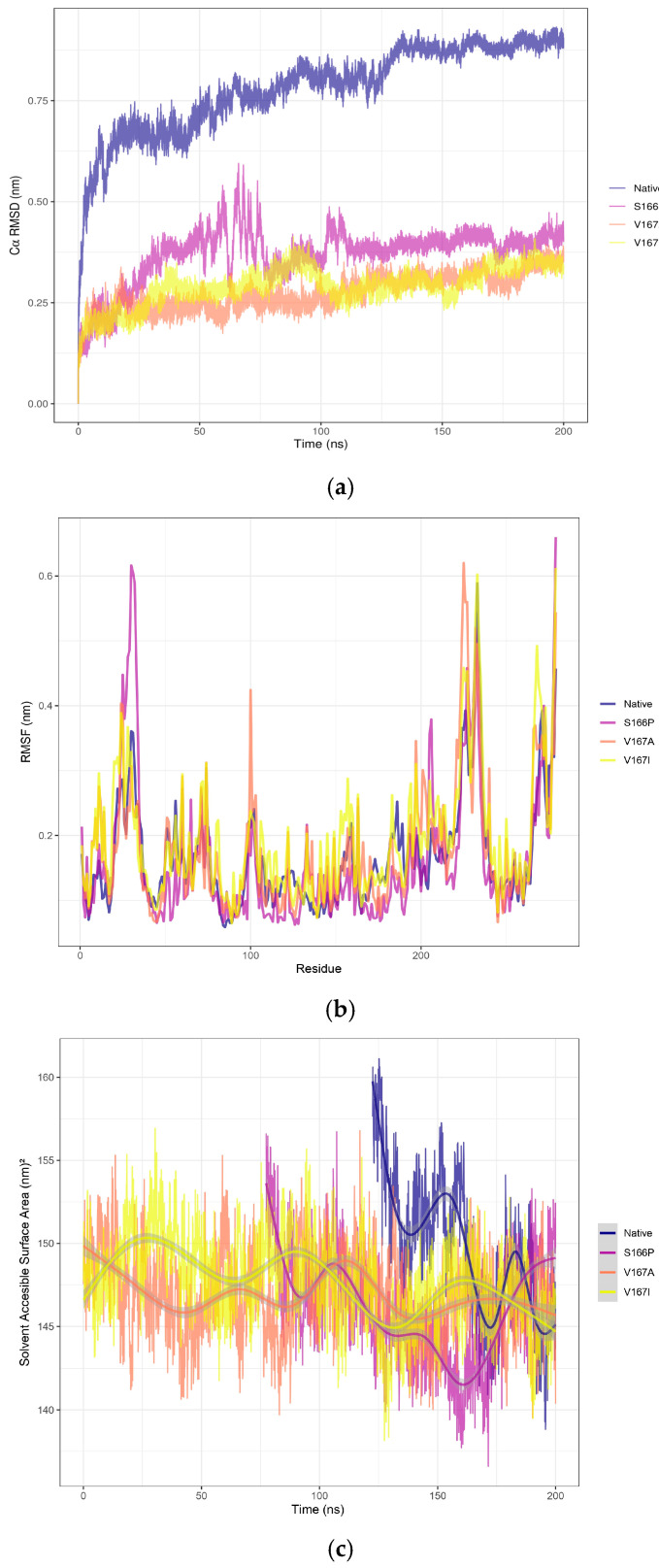
Molecular dynamics revealed that the S166P mutation significantly altered TetR-279 structure and flexibility, while V167A and V167I had minimal impact. Molecular dynamics analysis for overall conformational changes (backbone RMSD) and residue fluctuation in the putative TetR-279 protein. (**a**) The RMSD plot of the alpha carbons (Cα) as a function of time during a 200 ns molecular dynamics simulation for different variants of the TetR-279 protein: native, S166P, V167A, and V167I. (**b**) The root mean square fluctuation as a function of residue number for different variants of TetR-279 protein: native, S166P, V167A, and V167I. RMSF measures the flexibility of each residue in the protein structure over time during a molecular dynamics simulation. (**c**) The figure shows the evolution of SASA over time (in nanoseconds) for different protein variants throughout a 200 ns molecular dynamics simulation. The figure indicates that the S166P mutation has a more significant effect on the protein’s conformation, reducing its solvent-accessible surface area after compensating for proline residue-residue clashes. In contrast, the V167A and V167I mutations have less impact on the overall protein structure.

After structural refinement, given that TetR-279 was highlighted as a putative ligand-dependent regulator of the actinomycin D BGC, it was selected for MD simulations using GROMACS. A biological system was set up using periodic boundary conditions (PBCs) in a dodecahedron TIP3P water box, and a 200 ns simulation was executed at 288.15 K, as this temperature resembles the native environment of the *Streptomyces* strain. Root mean square deviation (RMSD) analysis showed that the structure reached stability after approximately 125 ns, indicating a conformationally stable state (Figure 3a). To evaluate the uniformity of conformational states during the stabilisation phase, clustering analysis was conducted using the GROMOS method with a 0.30 nm cutoff. This analysis identified a predominant cluster representing stabilised conformations, extending from 122.5 ns to 200 ns of the simulation. Following this, root mean square fluctuation (RMSF) analysis was performed on the stabilised cluster to pinpoint regions with high flexibility. The findings revealed that the C-terminal region exhibited the highest fluctuations, likely linked to structural compaction (Figure 3b). This observation was further corroborated by solvent-accessible surface area (SASA) analysis, which showed a gradual reduction in the accessible surface area over time, indicating progressive compaction of the structure (Figure 3c). To evaluate the structural quality of the representative snapshot obtained from the stabilised native TetR-279 model, Procheck was used. The analysis showed that 84.4% of residues were located in the most favoured regions, and importantly, no residues were found in disallowed regions of the Ramachandran plot (Figure S1). Although the percentage in favoured regions does not reach the typical standard of high-resolution crystal structures (commonly ≥90%), a combined 99.1% of residues falling within favoured and additionally allowed regions is considered acceptable for an in silico predictive model. Therefore, this structure was deemed suitable as a starting point for subsequent mutational analysis.

Based on the parameters defined in CRISPy-web and the protocol described by Tong [17], these correspond to the top-ranked random mutations, although their structural impact remains unknown. Three mutation models were developed to evaluate the impact of specific point mutations on TetR-279 stability, based on insights from MD simulations: S166P: A substitution introducing a proline, potentially affecting flexibility; V167A: A minor volume change that maintains hydrophobicity; and V167I: A conservative change affecting residue volume but preserving hydrophobicity. RMSD analysis (Figure 3a) showed that S166P caused the most significant perturbation, although the structure stabilised after 75 ns. In contrast, V167A and V167I showed minimal disturbances, with clustering analysis unable to distinguish them as distinct conformational states when compared with their starting structures, suggesting these mutations did not significantly impact structural dynamics. Additionally, RMSF analysis (Figure 3b) indicated that S166P caused significant fluctuations, particularly in the N-terminal region. Similarly, although V167A did not introduce major structural changes, it had a notable effect near the 100-residue region. In comparison, the V167I mutation resulted in only minor deviations relative to the other two. The SASA analysis (Figure 3c) confirmed that the V167A mutations did not notably affect structural stability, as the accessible surface area did not change significantly, whereas S166P led to substantial reorganisation—especially in the N-terminal region—as reflected by RMSF fluctuations. This effect aligns with the physicochemical changes introduced by the proline substitution, which reduces local backbone flexibility and causes broader structural reordering. Based on these findings, and drawing on reference data from CRISPR-based gene editing, three stabilised mutant models are proposed for future experimental validation to assess the structural consequences of TetR-279 mutations and their potential impact on its regulatory function in the actinomycin D biosynthetic pathway.

## 3. Discussion

The control of secondary metabolite production frequently involves various transcription factor families, although the specific regulators within each biosynthetic gene cluster can vary greatly between different species [18]. Although components of the TetR/AcrR TF family have been identified within the actinomycin D biosynthetic gene cluster, no reports currently confirm their direct role in regulating this cluster. Instead, in *Streptomyces antibioticus* ZS, a cluster-situated regulator, ActO, has been characterised and shown to belong to the LmbU family. ActO functions as a positive regulator of the entire actinomycin (act) cluster. Its deletion resulted in the complete loss of actinomycin D and V production, while overexpression increased their yields by 4.4-fold and 2.6-fold, respectively. Although ActO is not a TetR/AcrR family member, its function underscores the importance of cluster-specific regulatory elements in actinomycin biosynthesis [19]. A comprehensive review of the TetR family of regulators in actinobacteria has highlighted their diverse roles in the control of antibiotic biosynthesis, drug resistance, primary metabolism, and activation of silent biosynthetic gene clusters. While actinomycin D was not directly addressed, this review emphasises the broad functional relevance of TetR-like regulators in *Streptomyces* and their potential application in enhancing metabolite production or activating cryptic BGCs [20,24].

Furthermore, a TetR-like regulator (AalR) has been identified within the biosynthetic gene cluster for actinoallolides, macrolides with anti-trypanosomal activity produced by actinobacteria. Functional validation of AalR via heterologous expression confirmed its regulatory role, demonstrating that TetR-type regulators can be integral components of functional biosynthetic clusters in *Streptomyces* species [25]. The successful application of CRISPR/Cas9 to mutate TetR-family regulators demonstrates its potential for enhancing antibiotic production in *Streptomyces*. In *Streptomyces rimosus* M527, deletion of the TetR24 gene using CRISPR resulted in a 38% increase in rimocidin production, while overexpression reduced it by 40%. EMSA and DNase I footprinting confirmed that TetR24 binds directly to promoter regions of key biosynthetic genes (rimA and rimR2), acting as a direct repressor [21]. This study highlights the feasibility of using CRISPR to manipulate TetR regulators and opens the door for similar strategies in other biosynthetic clusters, including actinomycin D. While these findings were derived from a well-researched bacterium, there are no existing reports on the function of the TetR/AcrR transcription factor in Antarctic bacteria, nor are there any structural analyses that could facilitate the rational design of mutations to more effectively alter its activity.

Integrating bioinformatic tools into CRISPR-based genome editing processes has become crucial for predicting, validating, and enhancing genetic alterations, especially in complex organisms like *Streptomyces* [22]. A significant innovation is the creation of CRISPR-BEST, a base-editing system that circumvents DNA double-strand breaks. This system has been effectively utilised in *Streptomyces collinus* to introduce specific point mutations within the Kirromycin BGC. The method was confirmed through sequence-based bioinformatic analyses, including off-target prediction and mutation modelling, demonstrating its high precision and functional reliability [23]. In *Streptomyces fradiae*, a highly efficient CRISPR/Cas9n system was developed for targeted genome editing. Using high-fidelity Cas9 variants reduced cytotoxicity and enhanced editing specificity [26]. In addition to these methods, the EXPosition platform offers a new approach to designing CRISPR guide RNAs by predicting cutting efficiency and subsequent effects on gene expression, such as impacts on transcription, splicing, and translation. Although it has not yet been applied in *Streptomyces*, this strategy widely applies to bacteria with well-annotated genomes, providing valuable insights into how mutations might influence protein function [27]. Bioinformatic prediction tools were used to minimise and validate off-target effects, ensuring safe and precise modification of genes involved in neomycin biosynthesis [28]. Although the EXPosition platform boasts an innovative design, its predictive accuracy heavily relies on the presence of high-quality genome annotations. This is generally adequate for model organisms; however, numerous *Streptomyces* strains still lack comprehensive or consistent genomic annotations, especially in non-coding regions and regulatory elements [29]. This shortcoming can greatly affect the tool’s capacity to accurately predict splicing, transcriptional, and translational outcomes. Additionally, EXPosition does not conduct structural or functional simulations of the resulting proteins, which limits its ability to predict phenotypic or biochemical effects of CRISPR-induced mutations. Therefore, while the tool shows promise, its current version may offer limited predictive value for organisms with complex, partially annotated genomes like *Streptomyces*.

To fully grasp the biological significance of CRISPR-induced mutations, assessing their effects at the protein level is essential. Although many prediction tools focus on gene expression or sequence disruption, structural bioinformatics offers a more profound understanding of how mutations affect proteins [30]. Research by Chellapandi et al. [31] has demonstrated that even minor structural mutations can greatly impact enzymatic activity and virulence through molecular dynamics and modelling techniques. Likewise, Pires et al. [32] showed that structural simulations could predict over 80% of the phenotypic effects observed from mutagenesis, underscoring their role in connecting genotype to function. Consistent with these findings, our research employed GROMACS molecular dynamics to examine the TetR-279 protein, utilising RMSD, RMSF, SASA, and conformational clustering analyses. These methods enabled us to evaluate the stability and flexibility of the mutated protein and the importance of structural simulations in understanding CRISPR modifications. Amino acid changes like S166P, V167A, and V167I can profoundly impact protein structure and function by modifying essential physicochemical characteristics such as flexibility, volume, and hydrophobicity. The S166P change introduces proline, a residue known for its structural rigidity and tendency to disrupt α-helices by creating kinks. Yu et al. [33] demonstrated in their study on *Photinus pyralis* luciferase that inserting proline (D476P and H489P) in flexible regions increased thermostability but also risked impairing catalytic function due to limited backbone flexibility. This indicates that while the S166P mutation might enhance structural rigidity, it could also adversely affect dynamic regions crucial for protein activity.

On the other hand, the V167A and V167I mutations are conservative changes that preserve hydrophobicity but modify side chain volume. Matreyek et al. [34] found through large-scale mutagenesis across 14 proteins that such substitutions, especially involving residues like valine, alanine, and isoleucine, can subtly affect protein stability and folding, largely depending on the local structural context. While valine-to-alanine substitutions decrease side chain bulk, valine-to-isoleucine increases it, potentially influencing local packing interactions without significantly disrupting the hydrophobic core [35]. These findings highlight that even conservative or seemingly minor substitutions can lead to functionally significant outcomes. Their effects rely not only on the inherent properties of the substituted residues, but also on their positional context within secondary and tertiary structural elements, underscoring the importance of combining mutagenesis with structural and dynamic analyses to predict phenotypic consequences. These structural insights suggest that inhibition of TetR-279 through CRISPR-induced mutations or regulatory interference could release metabolic constraints imposed on the actinomycin cluster and potentially activate alternative or silent pathways involved in secondary metabolite production. This strategy could represent a novel route to induce biosynthetic diversity in extremophilic bacteria.

## 4. Materials and Methods

### 4.1. Identification and Structural Analysis of the Protein

In the genome of *Streptomyces* sp. So13.3 (CP048835.1), a BGC associated with the production of actinomycin D was discovered using AntiSMASH. This cluster includes two genes that code for TFs belonging to the TetR/AcrR subtype family: TetR-206 (Protein ID: QNA70898.1), a protein with 206 amino acids, and TetR-279 (Protein ID: QNA70921.1), a protein consisting of 279 amino acids. A BLAST search was conducted against the Protein Data Bank (PDB) to check for available crystallised structures, but none were found [36]. Consequently, homology modelling was carried out using AlphaFold [37], with the following UniProt identifiers: TetR-206 (UniProt: A0A5R9MB50) and TetR-279 (UniProt: A0A5R9M760), and the information regarding predicted domains for these proteins was retrieved from the Family and Domains section, based on annotations provided by InterProScan. Furthermore, SignalP 5.0 [38] was utilised to evaluate the presence of signal peptides and their potential role in subcellular localisation. The DP-Bind webserver (https://lcg.rit.albany.edu/dp-bind/help.html, accessed on 30 June 2024) was used to predict DNA-binding residues in TetR-279. Internally, it implements three machine learning methods: support vector machine (SVM), kernel logistic regression (KLR), and penalised logistic regression (PLR). The strict consensus assigns a binary “1” only to residues where all three methods agree, using an internal threshold specific to each model.

### 4.2. Cultivation Conditions of *Streptomyces* fildesensis So13.3

The strain *Streptomyces fildesensis* So13.3 was previously isolated from Antarctic soil samples and characterised through morphological and molecular analyses, as reported by Nuñez et al. [12]. For the current research, stock cultures of *Streptomyces fildesensis* So13.3 were initially cultivated on M2 agar plates at pH 7.0 and a temperature of 15 °C for one week. These cultures were then utilised in subsequent experimental assays, which included analysing growth under various nutritional conditions and evaluating gene expression (Table 2).

### 4.3. Gene Expression Analysis of TetR-279 Under Different Nutritional Conditions

To assess TetR-279 expression under varying nutritional conditions, bacterial cultures of *Streptomyces fildesensis* So13.3 were cultivated in three different media: IMA, ISP4, and YES, with M2 medium used as a control. Each bacterial culture was started from a single colony and inoculated into 1.5 mL of the respective media using a micro bioreactor system from Applikon Biotechnology (Delft, The Netherlands). For each type of medium, an uninoculated well served as a negative control. The cultures were incubated at 15 °C with shaking at 190 rpm for 7 days or until they reached optimal growth [39]. After incubation, total RNA was extracted using Trizol (Invitrogen, Waltham, MA, USA) following the manufacturer’s instructions. Quantitative real-time PCR (qPCR) was conducted using the Power SYBR^®^ Green RNA-to-CT™ 1-Step Kit (4389986, ThermoFisher, Waltham, MA, USA) on a LightCycler 96 system (Roche, Basel, Switzerland). Three biological replicates were examined, with each sample having two technical replicates. The relative expression levels of TetR-279 were calculated using the ^ΔΔCt^ method [40], with APRT (adenine phosphoribosyltransferase) mRNA serving as the internal reference gene for normalisation [41]. Specific primers were designed and used to amplify the *tetR-279* gene and the internal reference gene *APRT*. For *tetR-279*, the primers were Fw1 “CGAGTACACGCAGTTGGAGA”, Rv1: “GATGACACGACGGACCTTCA”. For *APRT*, the primers used were: Fw2: “GGAGCTCGGCTTCCTCAAG”, Rv2: “ACGATGATCAGTGCGTCCAG”. The results were analysed using GraphPad Prism version 9.5.0 (GraphPad Software Inc., San Diego, CA, USA). Comparisons between independent treatments were performed using one-way ANOVA.

### 4.4. sgRNA Design Targeting Transcriptional Regulators of the Actinomycin D Cluster

The sgRNA was designed by adhering to the approach outlined by Tong et al. [17]. To achieve this, genomic information from *Streptomyces* sp. So13.3, accessed via CRISPy web [42], was utilised alongside antiSMASH [43] to pinpoint the segment containing the actinomycin D biosynthetic gene cluster, situated between coordinates 461,186 and 529,693. The gene coding for the TetR/AcrR-type regulator (C8250_002330) was identified within this segment. Five sgRNAs were crafted, and the sequence deemed most appropriate was “AGAGCACGGAGATGACACGA”, with the associated PAM site “CGG”.

### 4.5. Homology Modelling, Refinement, and Molecular Dynamics Simulations

The models generated by AlphaFold were evaluated by examining their confidence scores to pinpoint regions of low resolution. Low-confidence structural loops (based on the pLDDT Alphafold internal score) were found between residues 1 and 14 in TetR-206, and between residues 18 and 37 in TetR-279. These areas were refined using GalaxyWeb Server [44], resulting in models used as starting structures for MD simulations. TetR-279 was chosen for MD simulations with GROMACS [45] due to its predicted C-terminal tetracyclin repressor-like motif, which can modulate this TF’s response via ligand binding, as reported for other members of the TetR TF family. The system was set up with PBC in a dodecahedron box. The GROMACS 2019 software suite was used for molecular dynamics simulations, adhering to the methodology established by Berendsen [45]. The energy minimisation procedure consisted of 5000 steps with an energy tolerance (emtol) value of 1000 kJ/mol/nm. Equilibration steps were divided into NVT and NPT phases, with temperature coupling to T = 288.15 K using the Berendsen thermostat. In the NPT equilibration, pressure coupling utilised the Berendsen barostat with an isotropic coupling scheme with a compressibility value of 4.5 × 10^−5^. Additionally, a restraint of 1000 kJ/mol/nm was applied to all alpha carbons along the x, y, and z axes to maintain their position during equilibration steps. Non-bonded interactions were computed using Lennard-Jones potentials for van der Waals forces, and long-range electrostatics were calculated using the Particle Mesh Ewald (PME) method with a cutoff of 1.0 nm. For the production run, electrostatic and nonbonded interactions were computed using the same method as in equilibration, but temperature and pressure coupling schemes were changed to V-rescale and Parrinello–Rahman, respectively. LINCS constraints for H-bonds were applied at each step, and a time step of 2 femtoseconds was used. Simulation visualisation was performed using the VMD 1.9.3 software [46]. Protein structure images were obtained using Protein Imager [47] and plots were generated using the ggplot2 R package [48]. An RMSD analysis was carried out to assess the structural stability over time. Clustering analysis was performed using the GROMOS method with a cutoff of 0.30 nm to identify dominant conformational states. The resulting clusters were examined to select the representative model of the stabilised conformations. Structural quality assessment was conducted using the SAVES v6.1 Procheck module (https://saves.mbi.ucla.edu/, accessed on 30 June 2024). To pinpoint regions with significant flexibility, RMSF analysis was carried out on the stabilised cluster, obtaining data related to each cluster timeframe. Furthermore, SASA analysis was performed to evaluate the degree of structural compaction and explore its possible relationship with RMSF variations. Modeller 10.5 [49] was employed to introduce amino acid mutations, and the resulting structures were then examined using molecular dynamics simulations conducted with GROMACS, as outlined in the previously described methodology.

## 5. Conclusions

This study provides substantial evidence of the biotechnological potential of *Streptomyces fildesensis* So13.3, an Antarctic strain containing a BGC related to the production of actinomycin D. Through an integrative approach combining gene expression analysis, structural modelling, molecular dynamics, and bioinformatic prediction, the role of TetR/AcrR family transcription factors as potential cluster regulators was thoroughly characterised. The differential expression of these regulators under specific nutritional conditions, particularly in ISP4 and IMA media, suggests that the metabolic environment may strongly influence cluster regulation. Structural analysis revealed key functional differences between the two identified TetR regulators, highlighting TetR-279 due to its C-terminal tetracycline-repressor-like domain, which may modulate its activity through interactions with metabolic ligands. Molecular dynamics simulations confirmed the overall structural stability of TetR-279 but also revealed significant alterations caused by point mutations—especially S166P, which notably affected the flexibility of the N-terminal region. This indicates that even conservative mutations like V167A and V167I can lead to localised structural effects. These findings underscore the importance of performing structural validation before CRISPR-based gene editing, allowing for better anticipation of the functional impact of mutations.

## Figures and Tables

**Table 1:** Regulatory proteins of the TetR/AcrR family identified in the actinomycin D biosynthetic cluster.

Locus Tag	Product	Length		Function
		NT	AA	
C8250_002175	TetR/AcrR family transcriptional regulator	621	206	Regulatory
C8250_002330	TetR/AcrR family transcriptional regulator	840	279	Regulatory

NT: Nucleotide; AA: Amino acid.

**Table 2:** Culture media and nutritional composition.

Medium	Components (Per Litre)
IMA	Yeast extract (4 g), Malt extract (10 g), Glucose (4 g), Mannitol (40 g)
YES	Sucrose (150 g), Yeast extract (20 g), MgSO_4_·7H_2_O (0.5 g), ZnSO_4_·7H_2_O (0.01 g), CuSO_4_·5H_2_O (0.005 g)
ISP-4	Starch (10 g), CaCO_3_ (2 g), (NH_4_)_2_SO_4_ (2 g), K_2_HPO_4_ (1 g), MgSO_4_·7H_2_O (1 g), NaCl (1 g), FeSO_4_·7H_2_O (1 mg), MnCl_2_·7H_2_O (1 mg), ZnSO_4_·7H_2_O (1 mg)
M2	Mannitol (40 g), Maltose (40 g), Yeast extract (10 g), K_2_HPO_4_ (2 g), MgSO_4_·7H_2_O (0.5 g), FeSO_4_·7H_2_O (0.01 g)

## Data Availability

Data is contained within the article and Supplementary Materials.

## Supplementary Materials

The following supporting information can be downloaded at: https://www.mdpi.com/article/10.3390/ijms26104839/s1.

## Abbreviations

The following abbreviations are used in this manuscript:

BGC	Biosynthetic Gene Cluster
CRISPR	Clustered Regularly Interspaced Short Palindromic Repeats
MD	Molecular Dynamics
RMSD	Root Mean Square Deviation
RMSF	Root Mean Square Fluctuation
SASA	Solvent Accessible Surface Area
TF	Transcription Factor
ISP4	International Streptomyces Project Medium 4
YES	Yeast Extract Sucrose Medium
HTH	Helix-Turn-Helix
SNP	Sodium Nitroprusside
LPS	Lipopolysaccharide
qPCR	Quantitative Polymerase Chain Reaction
PDB	Protein Data Bank
pLDDT	Predicted Local Distance Difference Test
VMD	Visual Molecular Dynamics
PBC	Periodic Boundary Condition
PME	Particle Mesh Ewald
NVT	Constant Number, Volume, Temperature Ensemble
NPT	Constant Number, Pressure, Temperature Ensemble
LINCS	Linear Constraint Solver
EMSA	Electrophoretic Mobility Shift Assay
DSB	Double-Strand Break
NRPS	Non-Ribosomal Peptide Synthetase
CRISPR-BEST	CRISPR Base Editing SysTem
EXPosition	EXpression Prediction from Sequence Mutations

## References

[1] Vaithinathan A.G., Vanitha A. (2018). WHO global priority pathogens list on antibiotic resistance: An urgent need for action to integrate One Health data. Perspect. Public Health.

[2] Nisa T.T., Nakatani D., Kaneko F., Takeda T., Nakata K. (2024). Antimicrobial resistance patterns of WHO priority pathogens isolated in hospitalized patients in Japan: A tertiary center observational study. PLoS ONE.

[3] Choudhury S., Medina-Lara A., Smith R. (2022). Antimicrobial resistance and the COVID-19 pandemic. Bull. World Health Organ..

[4] Núñez-Montero K., Barrientos L. (2018). Advances in Antarctic Research for Antimicrobial Discovery: A Comprehensive Narrative Review of Bacteria from Antarctic Environments as Potential Sources of Novel Antibiotic Compounds Against Human Pathogens and Microorganisms of Industrial Importance. Antibiotics.

[5] Thompson T.P., Gilmore B.F. (2024). Exploring halophilic environments as a source of new antibiotics. Crit. Rev. Microbiol..

[6] Coppola D., Lauritano C., Zazo G., Nuzzo G., Fontana A., Ianora A., Costantini M., Verde C., Giordano D. (2023). Biodiversity of UV-Resistant Bacteria in Antarctic Aquatic Environments. J. Mar. Sci. Eng..

[7] Lugg D.J., Roy C.R. (1999). Ultraviolet radiation and health effects in the Antarctic. Polar Res..

[8] Silva L.J., Crevelin E.J., Souza D.T., Lacerda-Júnior G.V., de Oliveira V.M., Ruiz A.L.T.G., Rosa L.H., Moraes L.A.B. (2020). Actinobacteria from Antarctica as a source for anticancer discovery. Sci. Rep..

[9] Benaud N., Edwards R.J., Amos T.G., D’Agostino P.M., Gutiérrez-Chávez C., Montgomery K., Nicetic I., Ferrari B.C. (2021). Antarctic desert soil bacteria exhibit high novel natural product potential, evaluated through long-read genome sequencing and comparative genomics. Environ. Microbiol..

[10] Lebedeva J., Jukneviciute G., Čepaitė R., Vickackaite V., Pranckutė R., Kuisiene N. (2021). Genome Mining and Characterization of Biosynthetic Gene Clusters in Two Cave Strains of *Paenibacillus* sp. Front. Microbiol..

[11] Russell A., Lacret R., Truman A. (2019). Genome-led discovery of novel microbial natural products. Access Microbiol..

[12] Núñez-Montero K., Lamilla C., Abanto M., Maruyama F., Jorquera M.A., Santos A., Martinez-Urtaza J., Barrientos L. (2019). Antarctic *Streptomyces fildesensis So13.3* strain as a promising source for antimicrobials discovery. Sci. Rep..

[13] Núñez-Montero K., Quezada-Solís D., Khalil Z.G., Capon R.J., Andreote F.D., Barrientos L. (2020). Genomic and Metabolomic Analysis of Antarctic Bacteria Revealed Culture and Elicitation Conditions for the Production of Antimicrobial Compounds. Biomolecules.

[14] Crits-Christoph A., Diamond S., Butterfield C.N., Thomas B.C., Banfield J.F. (2018). Novel soil bacteria possess diverse genes for secondary metabolite biosynthesis. Nature.

[15] Scherlach K., Hertweck C. (2021). Mining and unearthing hidden biosynthetic potential. Nat. Commun..

[16] Leal K., Rojas E., Madariaga D., Contreras M.J., Nuñez-Montero K., Barrientos L., Goméz-Espinoza O., Iturrieta-González I. (2024). Unlocking Fungal Potential: The CRISPR-Cas System as a Strategy for Secondary Metabolite Discovery. J. Fungi.

[17] Tong Y., Whitford C.M., Blin K., Jørgensen T.S., Weber T., Lee S.Y. (2020). CRISPR–Cas9, CRISPRi and CRISPR-BEST-mediated genetic manipulation in streptomycetes. Nat. Protoc..

[18] Park W., Woo J.-K., Shin J., Oh K.-B. (2017). nonG, a constituent of the nonactin biosynthetic gene cluster, regulates nocardamine synthesis in Streptomyces albus J1074. Biochem. Biophys. Res. Commun..

[19] Liang Y., Lu H., Tang J., Ye X., Wei Y., Liao B., Liu L., Xu H. (2025). ActO, a positive cluster-situated regulator for actinomycins biosynthesis in *Streptomyces antibioticus* ZS. Gene.

[20] Lei Y., Asamizu S., Ishizuka T., Onaka H. (2023). Regulation of Multidrug Efflux Pumps by TetR Family Transcriptional Repressor Negatively Affects Secondary Metabolism in *Streptomyces coelicolor* A3(2). Appl. Environ. Microbiol..

[21] Yu D., Lin H., Bechthold A., Yu X., Ma Z. (2025). RS24090, a TetR family transcriptional repressor, negatively affects the rimocidin biosynthesis in *Streptomyces rimosus* M527. Int. J. Biol. Macromol..

[22] Naeem M., Alkhnbashi O.S. (2023). Current Bioinformatics Tools to Optimize CRISPR/Cas9 Experiments to Reduce Off-Target Effects. Int. J. Mol. Sci..

[23] Tong Y., Whitford C.M., Robertsen H.L., Blin K., Jørgensen T.S., Klitgaard A.K., Gren T., Jiang X., Weber T., Lee S.Y. (2019). Highly efficient DSB-free base editing for streptomycetes with CRISPR-BEST. Proc. Natl. Acad. Sci. USA.

[24] Xu Y., Liu M., Zhao R., Pan Y., Wu P., Zhang C., Chi X., Zhang B., Wu H. (2024). TetR family regulator AbrT controls lincomycin production and morphological development in *Streptomyces lincolnensis*. Microb. Cell Fact..

[25] Inahashi Y., Shiraishi T., Také A., Matsumoto A., Takahashi Y., Ōmura S., Kuzuyama T., Nakashima T. (2018). Identification and heterologous expression of the actinoallolide biosynthetic gene cluster. J. Antibiot..

[26] Liu M.-S., Gong S., Yu H.-H., Jung K., Johnson K.A., Taylor D.W. (2019). Taylor, Basis for discrimination by engineered CRISPR/Cas9 enzymes. bioRxiv.

[27] Cohen S., Bergman S., Lynn N., Tuller T. (2024). A tool for CRISPR-Cas9 gRNA evaluation based on computational models of gene expression. bioRxiv.

[28] Yuan S. (2024). Mitigating the Off-target Effects in CRISPR/Cas9-mediated Genetic Editing with Bioinformatic Technologies. Trans. Mater. Biotechnol. Life Sci..

[29] Hwang S., Lee N., Jeong Y., Lee Y., Kim W., Cho S., Palsson B.O., Cho B.-K. (2019). Primary transcriptome and translatome analysis determines transcriptional and translational regulatory elements encoded in the *Streptomyces clavuligerus* genome. Nucleic Acids Res..

[30] Hussein Z.A., Al-Kazaz A.A. (2023). Bioinformatics Evaluation of Crisp2 Gene Snps and Their Impacts on Protein. Iraqi J. Agric. sciences.

[31] Chellapandi P. (2014). Structural-functional integrity of hypothetical proteins identical to ADPribosylation superfamily upon point mutations. Protein Pept. Lett..

[32] Pires D.E.V., Chen J., Blundell T.L., Ascher D.B. (2016). Ascher, In silico functional dissection of saturation mutagenesis: Interpreting the relationship between phenotypes and changes in protein stability, interactions and activity. Sci. Rep..

[33] Yu H., Zhao Y., Guo C., Gan Y., Huang H. (2015). The role of proline substitutions within flexible regions on thermostability of luciferase. Biochim. Biophys. Acta.

[34] Atsavapranee B., Sunden F., Herschlag D., Fordyce P.M. (2025). Quantifying protein unfolding kinetics with a high-throughput microfluidic platform. bioRxiv.

[35] Kellis J.T., Nyberg K., Fersht A.R. (1989). Energetics of complementary side-chain packing in a protein hydrophobic core. Biochemistry.

[36] Berman H.M., Westbrook J., Feng Z., Gilliland G., Bhat T.N., Weissig H., Shindyalov I.N., Bourne P.E. (2000). The Protein Data Bank. Nucleic Acids Res..

[37] Jumper J., Evans R., Pritzel A., Green T., Figurnov M., Ronneberger O., Tunyasuvunakool K., Bates R., Žídek A., Potapenko A. (2021). Highly accurate protein structure prediction with AlphaFold. Nature.

[38] Armenteros J.J.A., Tsirigos K.D., Sønderby C.K., Petersen T.N., Winther O., Brunak S., Von Heijne G., Nielsen H. (2019). SignalP 5.0 improves signal peptide predictions using deep neural networks. Nat. Biotechnol..

[39] Núñez-Montero K., Rojas-Villalta D., Hernández-Moncada R., Esquivel A., Barrientos L. (2023). Genome Sequence of *Pseudomonas* sp. Strain So3.2b, Isolated from a Soil Sample from Robert Island (Antarctic Specially Protected Area 112), Antarctic. Microbiol. Resour. Announc..

[40] Livak K.J., Schmittgen T.D. (2001). Analysis of Relative Gene Expression Data Using Real-Time Quantitative PCR and the 2^−ΔΔCT^ Method. Methods.

[41] Lin S., Zou Z., Zhou C., Zhang H., Cai Z. (2019). Transcriptome Analysis Reveals the Molecular Mechanisms Underlying Adenosine Biosynthesis in Anamorph Strain of Caterpillar Fungus. Biomed Res. Int..

[42] Blin K., Pedersen L.E., Weber T., Lee S.Y. (2016). CRISPy-web: An online resource to design sgRNAs for CRISPR applications. Synth. Syst. Biotechnol..

[43] Blin K., Shaw S., Steinke K., Villebro R., Ziemert N., Lee S.Y., Medema M.H., Weber T. (2019). antiSMASH 5.0: Updates to the secondary metabolite genome mining pipeline. Nucleic Acids Res..

[44] Afgan E., Baker D., Batut B., van den Beek M., Bouvier D., Čech M., Chilton J., Clements D., Coraor N., Grüning B.A. (2018). The Galaxy platform for accessible, reproducible and collaborative biomedical analyses: 2018 update. Nucleic Acids Res..

[45] Berendsen H.J., van der Spoel D., van Drunen R. (1995). GROMACS: A message-passing parallel molecular dynamics implementation. Comput. Phys. Commun..

[46] Humphrey W., Dalke A., Schulten K. (1996). VMD: Visual molecular dynamics. J. Mol. Graph..

[47] Tomasello G., Armenia I., Molla G. (2020). The Protein Imager: A full-featured online molecular viewer interface with server-side HQ-rendering capabilities. Bioinformatics.

[48] Wickham H. (2016). ggplot2.

[49] MODELLER A Program for Protein Structure Modeling Release 10.6, r12888. https://salilab.org/modeller/10.6/manual/?utm_source=chatgpt.com.

