# Supplementary material for: Structural Characterisation of TetR/AcrR Regulators in Streptomyces fildesensis So13.3: An In Silico CRISPR-Based Strategy to Influence the Suppression of Actinomycin D Production
Source: Int J Mol Sci. 2025 May 19;26(10):4839. doi: 10.3390/ijms26104839 (PMC12112392; doi:10.3390/ijms26104839)
Supplement: Supplementary file 1 [file ijms-26-04839-s001.zip › ijms-3587889-supplementary.pdf]

PROCHECK

Ramachandran Plot

saves

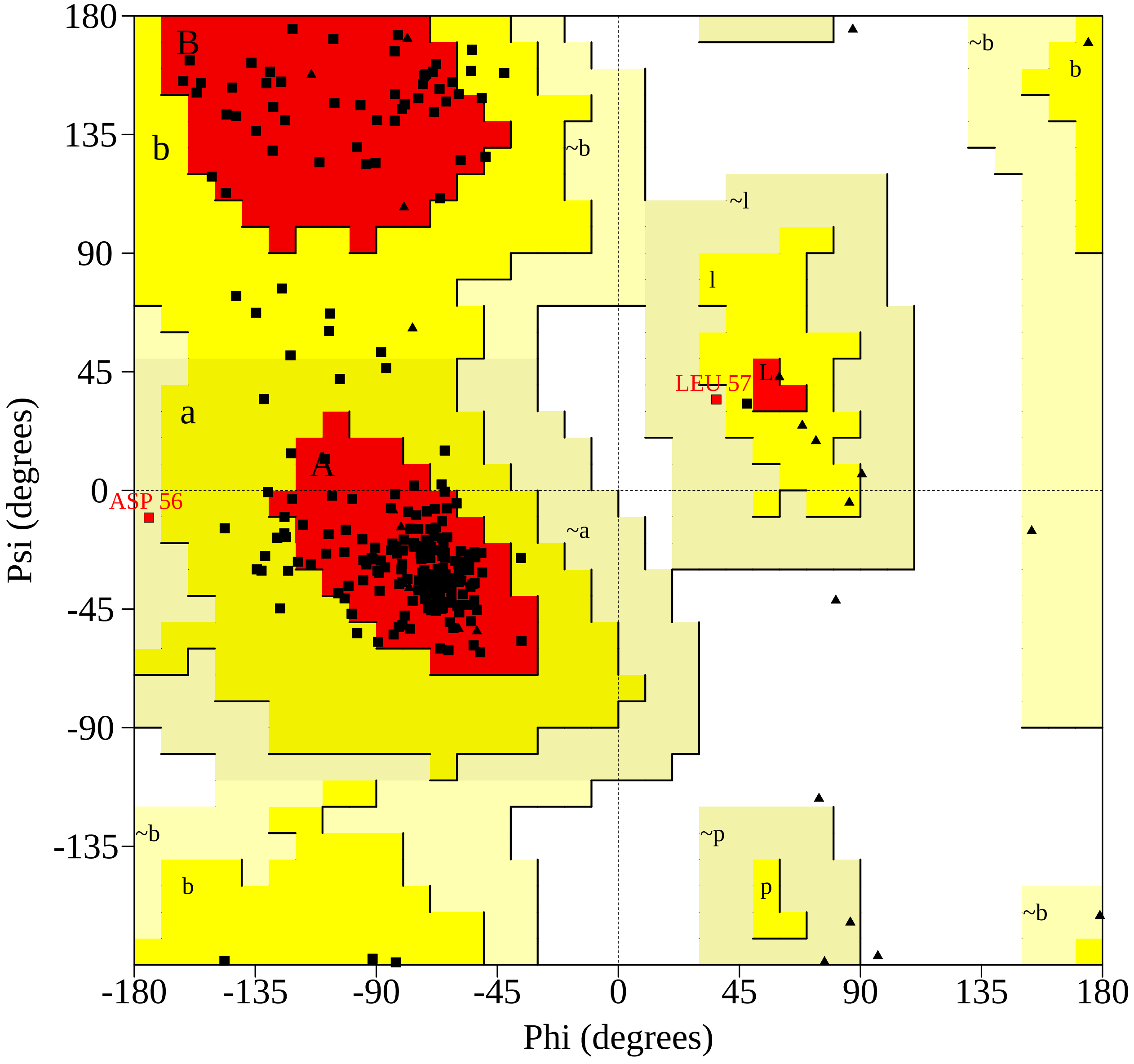

Plot statistics

Residues in most favoured regions [A,B,L]	200	84.7%
Residues in additional allowed regions [a,b,l,p]	34	14.4%
Residues in generously allowed regions [~a,~b,~l,~p]	2	0.8%
Residues in disallowed regions	0	0.0%
	----	------
Number of non-glycine and non-proline residues	236	100.0%
Number of end-residues (excl. Gly and Pro)	1	
Number of glycine residues (shown as triangles)	25	
Number of proline residues	17	

Total number of residues	279	

Based on an analysis of 118 structures of resolution of at least 2.0 Angstroms and R-factor no greater than 20%, a good quality model would be expected to have over 90% in the most favoured regions.
